# Predictors of atherosclerosis and dyslipidaemia among patients with thyroid disease: a cross-sectional analysis

**DOI:** 10.3389/fendo.2026.1814468

**Published:** 2026-08-07

**Authors:** Pronit Roy, Arindam Naskar, Mehebubar Rahman, Saikat Mandal, Arkadeep Dhali, Anindya Mukherjee, Omar Saafan

**Affiliations:** 1 Department of Tropical Medicine, School of Tropical Medicine, Kolkata, India; 2 Department of Endocrinology, Nutrition and Metabolic Diseases, School of Tropical Medicine, Kolkata, India; 3 Translational Medical Sciences, School of Medicine, University of Nottingham, Nottingham, United Kingdom; 4 NIHR Nottingham Biomedical Research Centre, Nottingham University Hospitals NHS Trust, Nottingham, United Kingdom; 5 Academic Gastroenterology, Sheffield Teaching Hospitals NHS Foundation Trust, Sheffield, United Kingdom; 6 Faculty of Health, Imperial College, London, England, United Kingdom; 7 Cardiology, Mid Yorkshire Teaching NHS Trust, Wakefield, United Kingdom; 8 Internal Medicine, Hull University Teaching Hospitals NHS Trust, Hull, United Kingdom

**Keywords:** atherosclerosis, autoimmune thyroid disease, dyslipidaemia, hyperthyroidism, hypothyroidism, lipid profile, metabolic disorder

## Abstract

**Background:**

Thyroid dysfunction is a risk factor for cardiovascular disease through its effects on lipid metabolism and arterial wall remodelling. However, the relative contributions of thyroid autoimmunity versus hormone deficiency to dyslipidaemia and atherosclerotic disease remain incompletely characterised in ageing populations.

**Objectives:**

To investigate the independent associations between thyroid dysfunction (overt hypothyroidism, subclinical hypothyroidism, overt hyperthyroidism, and subclinical hyperthyroidism) and early atherosclerotic markers, including lipid profiles, carotid intima-media thickness (CIMT), and to identify key predictors of hypercholesterolemia in an adult Indian population with thyroid dysfunctions.

**Methods:**

This cross-sectional observational study enrolled 82 consecutive adult patients with thyroid dysfunction from a tertiary care centre in eastern India. Patients receiving active thyroid hormone supplementation, antithyroid medication, or lipid-lowering treatment, including current statin therapy, were excluded. Thyroid and lipid measurements were performed using routine automated clinical laboratory methods, and participants underwent carotid ultrasound assessment. Multivariable binary logistic regression was performed to identify exploratory predictors of elevated total cholesterol (>200 mg/dL), with odds ratios, 95% confidence intervals and the area under the receiver operating characteristic curve (AUC) used to describe internal model performance. Consecutive eligible patients were recruited over a prespecified 12-month feasibility period; the sample size was based on available recruitment and supported by a *post-hoc* power/precision assessment for the principal correlation analyses.

**Results:**

Overt hypothyroidism was associated with higher CIMT and a greater burden of abnormal CIMT categories than the other thyroid-status groups. Total cholesterol was highest in the overt hypothyroid group, and TSH was positively correlated with CIMT in the overall cohort (Spearman rho = 0.65; p < 0.001). Peak systolic velocity was also highest in overt hypothyroidism. In exploratory multivariable logistic regression, log10(Anti-TPO) was associated with hypercholesterolaemia (OR 13.629, 95% CI: 1.174-190.489; p = 0.0421), whereas log10(TSH) was not independently associated after adjustment (OR 1.243, p = 0.6904). The wide confidence interval indicates substantial imprecision and the finding requires external validation. The internally assessed logistic model showed acceptable discrimination (AUC 0.834, 95% CI: 0.748-0.920), and Anti-TPO was positively correlated with CIMT (rho = 0.60; p < 0.001).

**Conclusion:**

Overt hypothyroidism was associated with early atherosclerotic changes and dyslipidaemia in this exploratory single-centre cohort. Anti-TPO antibodies correlated with CIMT and were associated with elevated total cholesterol in an adjusted model, but the imprecision of the estimate and cross-sectional design mean that these findings should be considered hypothesis-generating and require prospective external validation before clinical implementation.

## Introduction

Thyroid dysfunction is common worldwide, but prevalence estimates vary across populations, iodine status, age, sex and diagnostic thresholds. Contemporary epidemiological studies continue to show a substantial burden of hypothyroid and hyperthyroid disorders, with subclinical disease forming an important proportion of detected thyroid dysfunction (1–3). Beyond effects on metabolic rate, thyroid dysfunction has systemic consequences, including lipid metabolism, vascular endothelial function, and atherosclerotic disease progression (1–3).

Hypothyroidism characteristically produces an atherogenic lipid profile with elevated total cholesterol, low-density lipoprotein cholesterol (LDL-C), and triglycerides, often accompanied by reduced high-density lipoprotein cholesterol (HDL-C) (4). Conversely, hyperthyroidism typically results in lower lipid concentrations, though it may paradoxically confer cardiovascular risk through haemodynamic perturbations and pro-inflammatory mechanisms (4, 5). Whether these lipid alterations serve primarily as biomarkers of atherosclerotic progression or constitute mechanistic drivers of arterial wall disease remains an important clinical question.

Thyroid hormones influence lipid metabolism through coordinated hepatic and peripheral mechanisms. Experimental data link thyroid hormone signalling to SREBP-2-mediated regulation of LDL receptor pathways and cholesterol biosynthesis, while clinical studies show that hypothyroidism is associated with reduced LDL clearance and elevated LDL-C and total cholesterol. Thyroid hormones also influence triglyceride-rich lipoprotein metabolism, hepatic cholesterol-to-bile acid conversion, and apolipoprotein pathways; these mechanisms help explain why lipid abnormalities are common in hypothyroid states (4, 6).

Thyroid dysfunction may also accelerate atherosclerosis through lipid-independent pathways. Thyroid hormone abnormalities and autoimmune thyroid disease have been linked to vascular dysfunction, oxidative stress and low-grade inflammation (5, 7–9). Autoimmune thyroid disease, predominantly Hashimoto’s thyroiditis, may perpetuate chronic immune activation, as reflected clinically by elevated anti-thyroid peroxidase (Anti-TPO) antibody levels. In some cohorts, Anti-TPO titres have been associated with surrogate vascular markers, including carotid arterial wall thickening, although their independent value for predicting clinical cardiovascular events remains uncertain (7, 8).

Carotid intima-media thickness (CIMT), assessed by high-resolution ultrasonography, is an established surrogate marker of subclinical atherosclerosis and has been associated with future cardiovascular events (10–12). A previous systematic review and meta-analysis reported that each 0.1 mm increase in CIMT was associated with higher future myocardial infarction and stroke risk (11). Systematic reviews and meta-analyses have also reported an association between subclinical hypothyroidism and increased CIMT, although causality and treatment implications remain uncertain (13, 14).

The relative contributions of thyroid hormone deficiency versus thyroid autoimmunity to atherosclerotic progression remain incompletely characterised, particularly in ageing populations and non-European cohorts. This cross-sectional study investigated associations between thyroid dysfunction across the overt and subclinical spectrum and early atherosclerotic markers, including lipid profiles and CIMT, in an adult Indian population. A secondary objective was to explore whether thyroid hormone status or Anti-TPO antibodies were independently associated with hypercholesterolaemia after adjustment for selected clinical and vascular variables.

## Methods

### Study design and population

This cross-sectional observational study was conducted at the School of Tropical Medicine, Kolkata, India, over a 12-month period. The study enrolled 82 consecutive patients aged >=18 years who presented to the outpatient and inpatient departments with thyroid dysfunction, as confirmed by clinical examination and laboratory findings. Consecutive recruitment was used to reduce investigator selection bias within the eligible single-centre clinical population. Inclusion criteria encompassed patients of either sex with documented thyroid dysfunction. Exclusion criteria included age <18 years, current use of medications affecting lipid profiles (including statins or other lipid-lowering therapy, thiazide diuretics, protease inhibitors, and prolonged steroid therapy), pre-existing dyslipidaemia from non-thyroidal causes, pregnancy, active thyroid hormone supplementation or antithyroid medication use, and inability to provide informed consent. Demographic information, clinical history and medication exposure were recorded at enrolment using a standardised data collection form.

Eligibility was confirmed before enrolment through clinical assessment, thyroid function tests and treatment history. Patients receiving thyroid-directed treatment were excluded because hormone replacement or antithyroid therapy could modify both biochemical thyroid status and lipid measures. Current statin or other lipid-lowering therapy users were excluded to minimise treatment-related confounding of lipid outcomes. These exclusions improve the internal validity of the biochemical analyses but reduce generalisability to treated populations with thyroid disease.

### Sample size and power considerations

This was an exploratory, single-centre cross-sectional study using consecutive recruitment over 12 months. No formal *a priori* sample size calculation was performed because the eligible population was determined by the fixed study period and strict exclusion criteria. However, the final sample of 82 participants provided adequate precision for the principal overall correlation analyses: using a two-sided alpha of 0.05, n=82 provides approximately 80% power to detect a correlation coefficient of about 0.30 and more than 90% power to detect correlations of about 0.35-0.40. The observed correlations between TSH and CIMT (Spearman rho=0.65) and Anti-TPO and CIMT (rho=0.60), therefore, exceeded the minimum effect size detectable with acceptable power. By contrast, subgroup analyses and multivariable logistic regression were considered exploratory because some thyroid-status groups were small and the number of outcome events was limited; therefore, odds ratios are reported with 95% confidence intervals and interpreted with caution.

### Measurements and assessments

#### Thyroid function tests

Serum TSH, free T3 (pg/mL), free T4 (ng/dL), and anti-thyroid peroxidase antibodies (Anti-TPO; IU/mL) were measured in the institutional clinical laboratory using routine automated electrochemiluminescence immunoassay methods on the cobas^®^ e 601 immunoassay module within the cobas^®^ 6000 analyser series platform (Roche Diagnostics). Patients were classified into four thyroid-status categories using laboratory-specific reference intervals and standard clinical definitions: (1) overt hypothyroidism, defined as elevated TSH with low free T4; (2) subclinical hypothyroidism, defined as elevated TSH with normal free T4; (3) overt hyperthyroidism, defined as suppressed TSH with elevated free T3 and/or free T4; and (4) subclinical hyperthyroidism, defined as suppressed TSH with normal free T3 and free T4. Anti-TPO positivity was defined as >35 IU/mL according to the laboratory reference threshold used in the study centre.

#### Lipid profiling

Fasting venous blood samples (5 mL) were collected and processed to obtain serum for lipid analysis. Total cholesterol, directly measured LDL-C, HDL-C, and triglycerides were measured in the institutional clinical laboratory using routine automated enzymatic colorimetric methods on the cobas^®^ c 501 clinical chemistry module within the cobas^®^ 6000 analyser series platform (Roche Diagnostics). Results were expressed in mg/dL. Hypercholesterolaemia, the dependent variable for logistic regression, was defined *a priori* as total cholesterol >200 mg/dL.

#### Carotid intima-media thickness

High-resolution B-mode ultrasonography was performed to assess CIMT of the common carotid artery bilaterally, with measurements expressed in centimetres. CIMT categories were defined as: normal (<0.07 cm), mild (0.07-0.09 cm), moderate (0.09-0.11 cm), and severe (>0.11 cm). Peak systolic velocity (cm/s) was recorded as an additional haemodynamic parameter (12). Participants were examined in the supine position, and the right and left common carotid arteries were assessed. The mean participant-level CIMT was used for analysis. Measurements were taken from clearly visualised arterial-wall interfaces, avoiding focal plaque where possible, and PSV was recorded during the same carotid assessment.

#### Ethical clearance

The study was approved by the Institutional Ethics Committee (CREC-STM/2022-D15). Each individual participating in the study provided informed written consent.

### Statistical analysis

Descriptive statistics were calculated with continuous variables expressed as mean +/- standard deviation and categorical variables as counts and percentages. Between-group comparisons used the Kruskal-Wallis test for continuous variables and the chi-square or Fisher’s exact test for categorical variables. Spearman’s correlation was the primary approach because TSH, Anti-TPO, and several lipid variables were non-normally distributed, and monotonic rather than strictly linear relationships were expected. Pearson correlation was reported only as a supportive analysis after log10 transformation of TSH, to describe the linear association on the transformed scale; it was not used to infer an exponential relationship. Logistic regression was performed to identify exploratory predictors of elevated cholesterol levels. Linear regression of TSH, adjusted for age and sex, was evaluated for its ability to predict CIMT. Statistical significance was set at two-tailed p<0.05. All analyses were performed using R statistical software. Variables for the multivariable logistic regression were selected *a priori* on clinical grounds and included age, sex, CIMT, log10(TSH), and log10(Anti-TPO). Stepwise selection was not used. TSH and Anti-TPO were log10-transformed to address right-skewed distributions and reduce the influence of extreme values. Model performance was assessed using the area under the receiver operating characteristic curve, with pseudo-R2, AIC and BIC used to describe model fit. Complete-case analysis was used for the prespecified thyroid, lipid and CIMT variables; no imputation was required for these primary variables. Given the modest sample size, all multivariable estimates were interpreted as exploratory and hypothesis-generating rather than definitive.

## Results

### Study population characteristics

The cohort comprised 82 patients with thyroid dysfunction. Participants were distributed across overt hypothyroidism, subclinical hypothyroidism, overt hyperthyroidism and subclinical hyperthyroidism groups. Thyroid function tests showed the expected biochemical pattern across these categories, and Anti-TPO positivity was most frequent in the hypothyroid spectrum. Detailed biochemical and lipid values are provided in **Table 1**; carotid ultrasound findings are provided separately in **Table 2**.

**Table 1:** Baseline demographic, thyroid and lipid characteristics by thyroid status.

Variable	Overt hypothyroidism (n=41)	Subclinical hypothyroidism (n=12)	Overt hyperthyroidism (n=18)	Subclinical hyperthyroidism (n=11)	P-value
Age (years)	46.98 +/- 8.45	56.58 +/- 9.09	40.17 +/- 7.37	48.73 +/- 7.21	<0.01
Sex - male, n (%)	23 (56.1%)	8 (66.7%)	10 (55.6%)	6 (54.5%)	0.916
Sex - female, n (%)	18 (43.9%)	4 (33.3%)	8 (44.4%)	5 (45.5%)	0.916
TSH (mIU/L)	41.03 +/- 57.01	7.86 +/- 1.07	0.19 +/- 0.27	0.36 +/- 0.06	<0.01
Free T4 (ng/dL)	0.52 +/- 0.07	1.18 +/- 0.10	3.81 +/- 1.20	1.19 +/- 0.14	<0.01
Free T3 (pg/mL)	1.02 +/- 0.10	3.29 +/- 0.52	10.61 +/- 2.64	2.91 +/- 0.66	<0.01
Anti-TPO (IU/mL)	321.41 +/- 178.86	42.08 +/- 6.32	24.72 +/- 7.18	27.73 +/- 9.12	<0.01
TPO positive (>35 IU/mL), n (%)	41 (100.0%)	11 (91.7%)	1 (5.6%)	2 (18.2%)	0.0000
Triglycerides (mg/dL)	257.17 +/- 56.33	231.08 +/- 74.25	197.89 +/- 78.47	239.45 +/- 69.07	0.0591
Total cholesterol (mg/dL)	273.63 +/- 72.63	181.42 +/- 86.66	203.78 +/- 97.22	183.73 +/- 88.25	<0.01
HDL-C (mg/dL)	41.17 +/- 7.52	41.33 +/- 8.05	38.83 +/- 5.98	41.45 +/- 8.36	0.7309
LDL-C (mg/dL)	180.15 +/- 48.35	176.42 +/- 51.73	182.00 +/- 47.93	179.91 +/- 50.03	0.9997

Continuous variables are presented as mean +/- SD; categorical variables are presented as n (%). P-values were calculated using Kruskal-Wallis tests for continuous variables and chi-square/Fisher exact tests for categorical variables. The large SDs for TSH, Anti-TPO, and some lipid variables reflect the biologically heterogeneous thyroid status groups and right-skewed biomarker distributions; therefore, TSH and Anti-TPO were log10-transformed in regression analyses.

**Table 2:** Carotid ultrasound findings by thyroid status.

Variable	Overt hypothyroidism (n=41)	Subclinical hypothyroidism (n=12)	Overt hyperthyroidism (n=18)	Subclinical hyperthyroidism (n=11)	P-value
CIMT (cm)	0.09 +/- 0.02	0.06 +/- 0.01	0.06 +/- 0.01	0.06 +/- 0.01	<0.01
Peak systolic velocity (cm/s)	139.98 +/- 42.35	91.33 +/- 9.51	96.50 +/- 20.55	88.18 +/- 9.91	<0.01
CIMT category - normal, n (%)	0 (0.0%)	11 (91.7%)	14 (77.8%)	10 (90.9%)	<0.01
CIMT category - mild, n (%)	26 (63.4%)	1 (8.3%)	3 (16.7%)	1 (9.1%)	
CIMT category - moderate, n (%)	12 (29.3%)	0 (0.0%)	1 (5.6%)	0 (0.0%)	
CIMT category - severe, n (%)	3 (7.3%)	0 (0.0%)	0 (0.0%)	0 (0.0%)	

CIMT, carotid intima-media thickness. Continuous variables are presented as mean +/- SD; categorical variables are presented as n (%).

### Lipid profile abnormalities across thyroid states

#### Total cholesterol and triglycerides

Total cholesterol differed significantly among thyroid-status groups, with the highest level in the overt hypothyroidism group. Triglycerides showed a between-group trend but did not meet statistical significance. Detailed values are provided in **Table 1**.

#### LDL-C and HDL-C

LDL-C and HDL-C did not differ significantly across thyroid-status groups. Detailed values are provided in **Table 1**.

### Carotid intima-media thickness and atherosclerotic burden

CIMT differed significantly across thyroid-status groups, with the highest mean CIMT and the greatest proportion of abnormal CIMT categories observed in overt hypothyroidism. Subclinical hypothyroidism, overt hyperthyroidism and subclinical hyperthyroidism groups showed lower CIMT values and a higher proportion of normal CIMT classifications. Detailed continuous and categorical ultrasound values are shown in **Table 2**.

Overall, 57.3% of the cohort had abnormal CIMT categories. Peak systolic velocity differed significantly across groups and was highest in overt hypothyroidism. Detailed PSV values are provided in **Table 2**.

### Correlation between thyroid function and atherosclerotic markers

#### TSH-CIMT correlation

**Figure 1:**
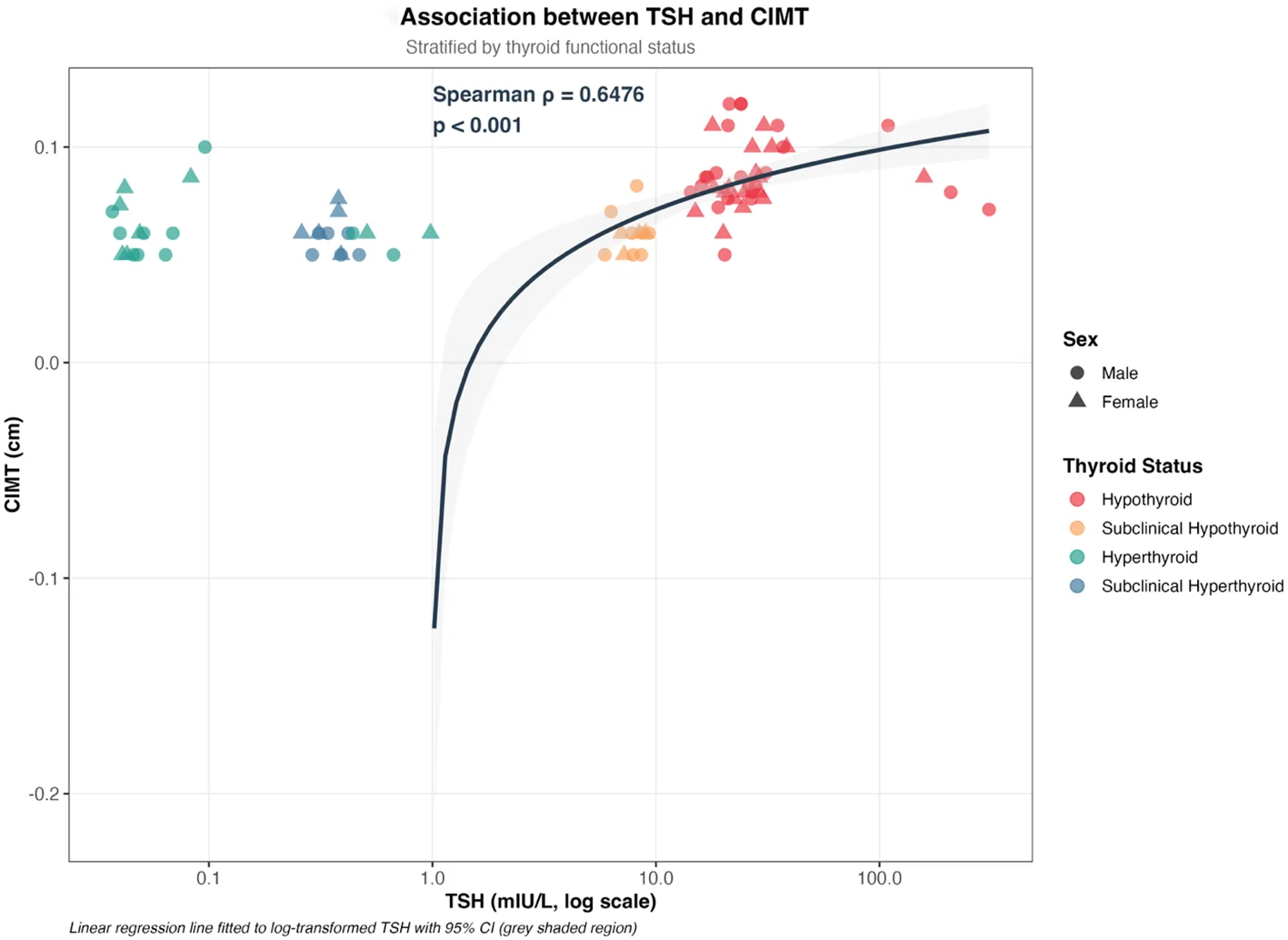
Association between thyroid-stimulating hormone (TSH) and carotid intima-media thickness (CIMT) stratified by thyroid functional status. The scatter plot illustrates the relationship between log-transformed TSH concentration (x-axis, mIU/L, logarithmic scale) and CIMT measurement (y-axis, cm) in the overall study cohort (n=82). Individual data points are colour-coded according to thyroid functional status: hypothyroidism (red), subclinical hypothyroidism (yellow), hyperthyroidism (green), and subclinical hyperthyroidism (blue). Biological sex is denoted by marker shape: circles represent male participants; triangles represent female participants. A linear regression line fitted to log-transformed TSH data is shown, with the corresponding 95% confidence interval (grey-shaded region).

TSH levels were positively correlated with CIMT in the overall cohort (Spearman rho = 0.65; p < 0.001). Pearson correlation using log10-transformed TSH was also reported as a supportive transformed-scale analysis (r = 0.53; p < 0.001) (**Figure 1**, **Table 3A**). This lower Pearson coefficient should not be interpreted as evidence of a stronger or exponential relationship. When stratified by thyroid status, correlations were weak within individual groups (Spearman rho range -0.23 to 0.22), with p-values shown in **Table 3B**.

**Table 3:** Summary of correlations with CIMT.

A. Overall correlation				
Parameter	Correlation type	Coefficient	P-value	Rank
TSH	Spearman ρ	0.6476	< 0.001	1
Anti-TPO	Spearman ρ	0.5981	< 0.001	2
Log_10_(TSH)	Pearson r	0.5312	< 0.001	3
Total Cholesterol	Spearman ρ	0.2836	0.0098	4
TSH (raw Pearson)	Pearson r	0.2512	0.0228	5
LDL-C	Spearman ρ	0.1450	0.1937	6
HDL-C	Spearman ρ	-0.0395	0.7249	7
Triglycerides	Spearman ρ	0.0359	0.7491	8
B. Correlation by thyroid status				
Thyroid status	N	Spearman rho	Approximate p-value	
Overt hypothyroidism	41	0.2207	0.165	
Subclinical hypothyroidism	12	0.1257	0.697	
Overt hyperthyroidism	18	-0.0206	0.935	
Subclinical hyperthyroidism	11	-0.2315	0.494	

#### Anti-TPO-CIMT correlation

Anti-TPO antibody levels were positively correlated with CIMT in the overall cohort (Spearman rho = 0.60; p < 0.001) (**Table 3A**).

#### Lipid parameter correlations

**Figure 2:**
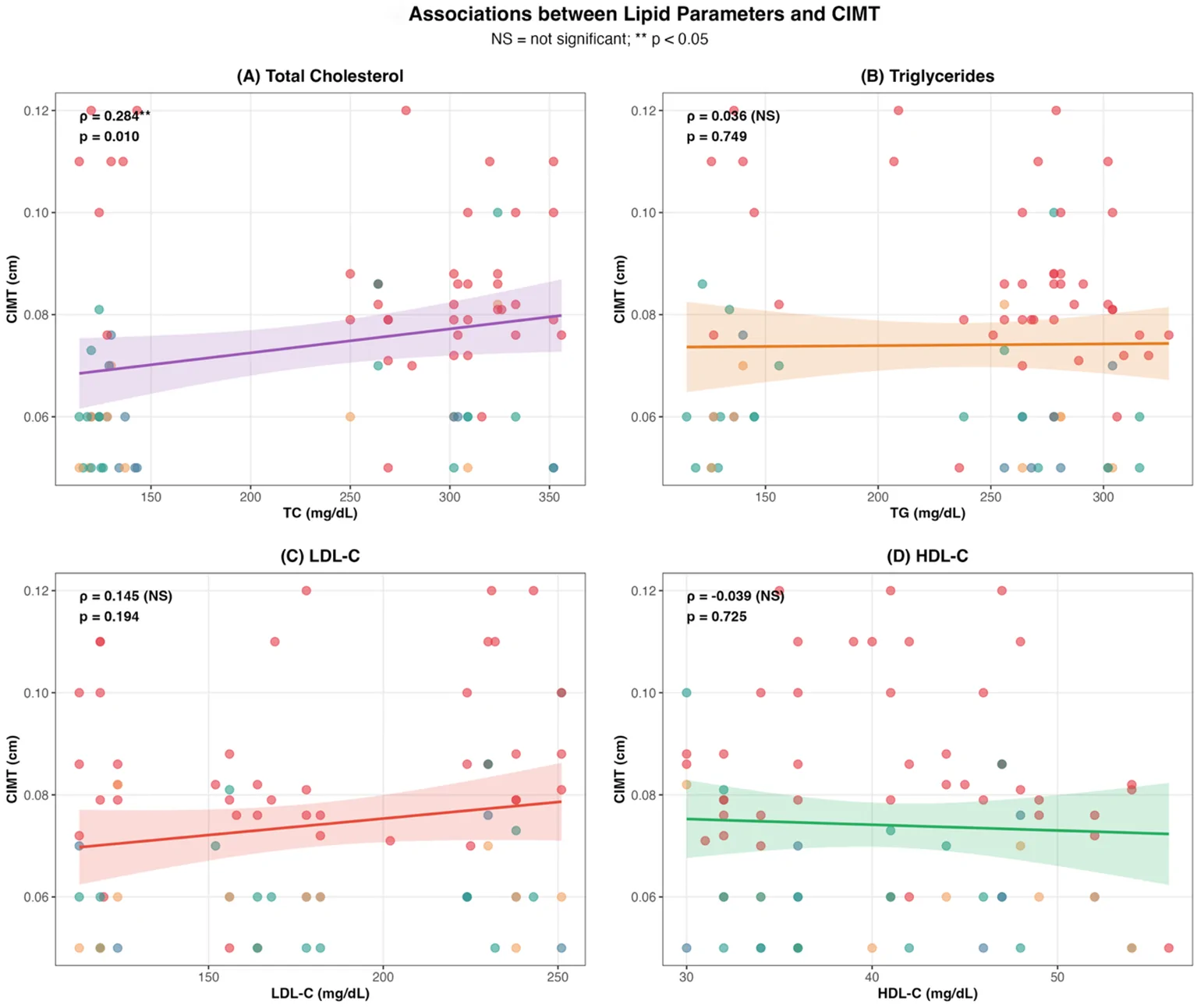
Associations between lipid parameters and carotid intima-media thickness (CIMT). Four-panel scatter plot depicting bivariate relationships between serum lipid concentrations and CIMT measurements. **(A)** Total cholesterol (TC) versus CIMT: Spearman ρ = 0.284, p = 0.010 (significant). **(B)** Triglycerides (TG) versus CIMT: Spearman ρ = 0.036, p = 0.749 (not significant). **(C)** Low-density lipoprotein cholesterol (LDL-C) versus CIMT: Spearman ρ = 0.145, p = 0.194 (not significant). **(D)** High-density lipoprotein cholesterol (HDL-C) versus CIMT: Spearman ρ = −0.039, p = 0.725 (not significant). Linear regression lines (coloured to match each lipid parameter) with 95% confidence intervals (shaded regions) are displayed in each panel.

A modest positive correlation was observed between total cholesterol and CIMT (rho = 0.28, p = 0.0098). LDL-C, triglycerides and HDL-C did not significantly correlate with CIMT (**Figure 2**, **Table 3A**).

### Independent predictors of atherosclerotic disease

**Figure 3:**
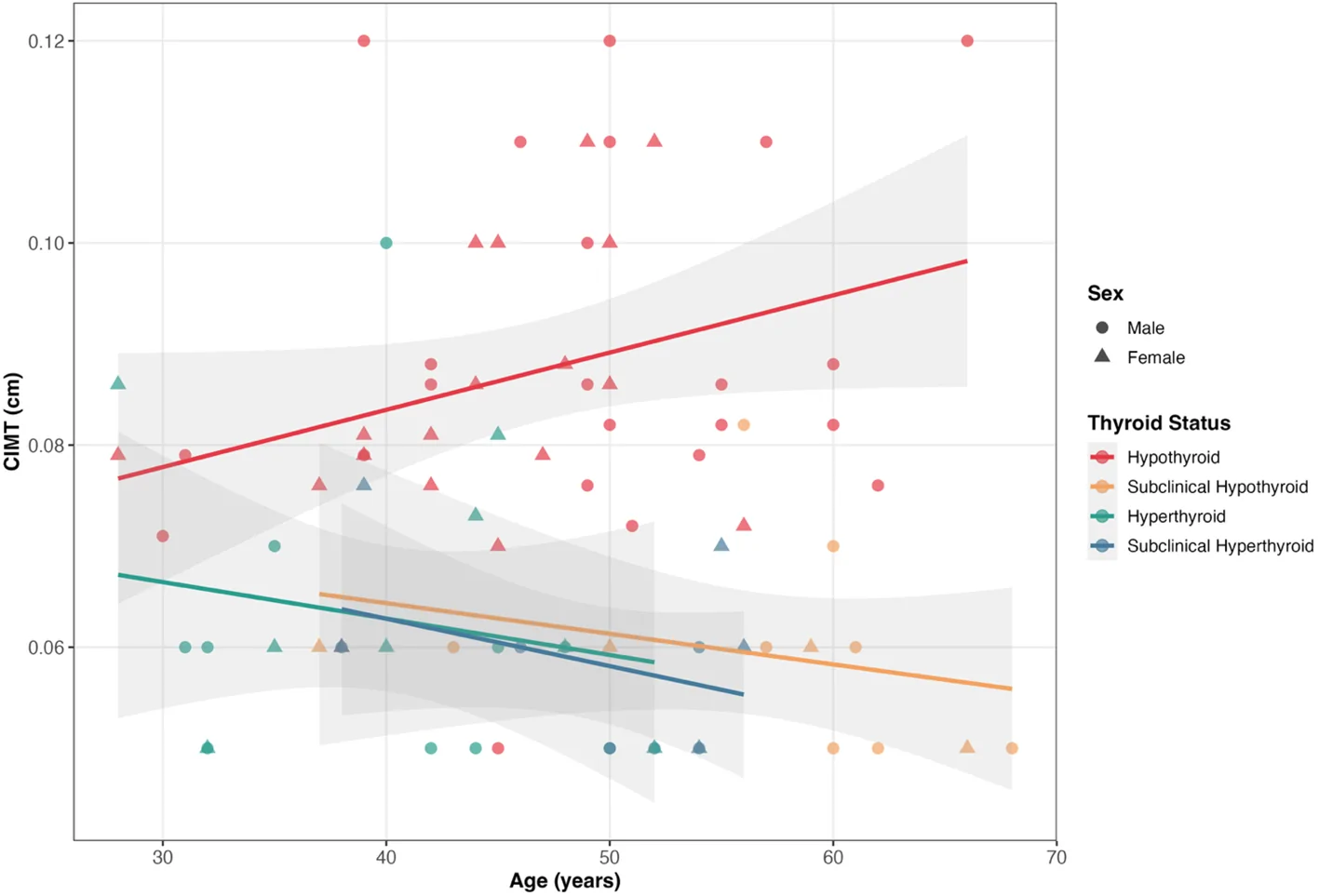
Relationship between age and carotid intima-media thickness (CIMT) stratified by thyroid functional status. Scatter plot displaying CIMT measurements (y-axis, cm) across the age spectrum (x-axis, years) with data points colour-coded by thyroid status (red, hypothyroidism; orange, subclinical hypothyroidism; teal, hyperthyroidism; blue, subclinical hyperthyroidism) and stratified by biological sex (circles, male; triangles, female). Linear regression lines with 95% confidence intervals (shaded regions) are displayed for each thyroid status category.

Adjusted linear regression showed that TSH level (beta = 0.000118, SE = 0.000049, p < 0.05) remained positively associated with CIMT after adjustment for age and sex, explaining 6.6% of CIMT variance (R2 = 0.066; adjusted R2 = 0.030) (**Table 4**). **Figure 3** provides a descriptive visual summary of age, CIMT and thyroid functional status.

**Table 4:** Adjusted linear regression (CIMT ~ TSH + age + sex).

Variable	Estimate	Std. Error	t value	P-value
Intercept	0.063163	0.012221	5.168	<0.01
TSH (mIU/L)	0.000118	0.000049	2.407	0.01846
Age (years)	0.000161	0.000237	0.677	0.50015
Female Sex	0.001771	0.004395	0.403	0.68807

Multiple R²: 0.0663 | Adjusted R²: 0.0303.

### Independent predictors of hypercholesterolemia

**Figure 4:**
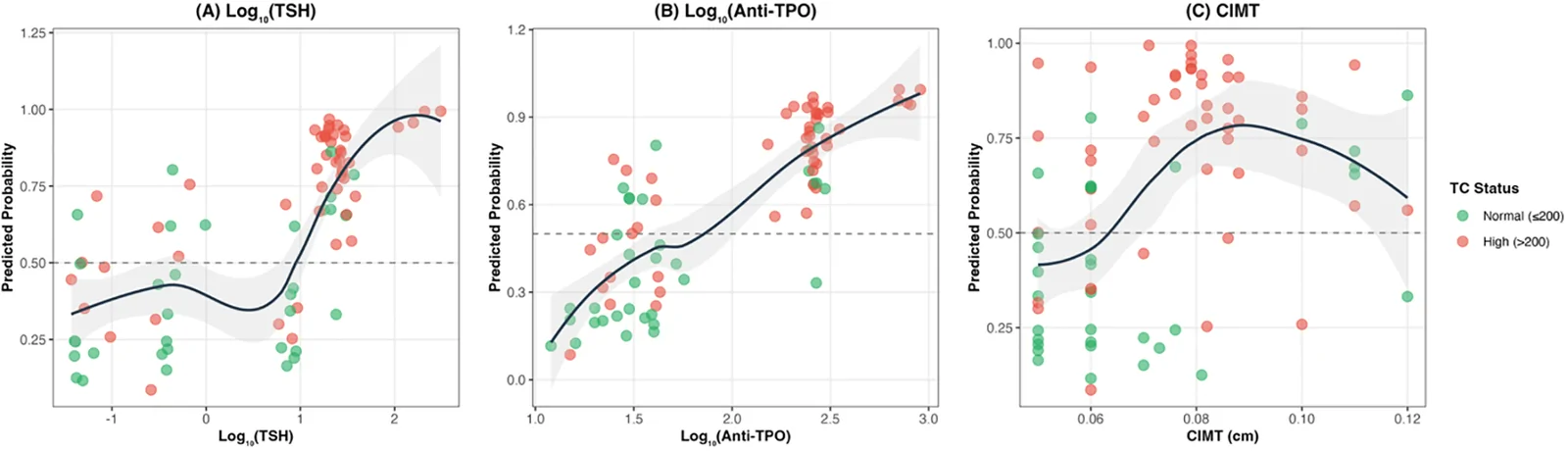
Predicted probability of hypercholesterolaemia across thyroid function, autoimmunity, and atherosclerotic markers. Three-panel figure presenting smoothed estimates of the probability of high total cholesterol (TC >200 mg/dL) across candidate predictors. Data points represent individual participants and are colour-coded by observed TC status: normal (≤200 mg/dL, green) versus high (>200 mg/dL, red). The solid line represents a LOESS-smoothed trend with a 95% confidence band (shaded region). **(A)** log_10_-transformed TSH; **(B)** log_10_-transformed anti-TPO (thyroid peroxidase antibody); **(C)** carotid intima-media thickness (CIMT, cm). Dashed horizontal lines indicate the 0.5 probability threshold. These plots are descriptive and should be interpreted alongside the multivariable logistic regression results (**Table 5**).

**Figure 5:**
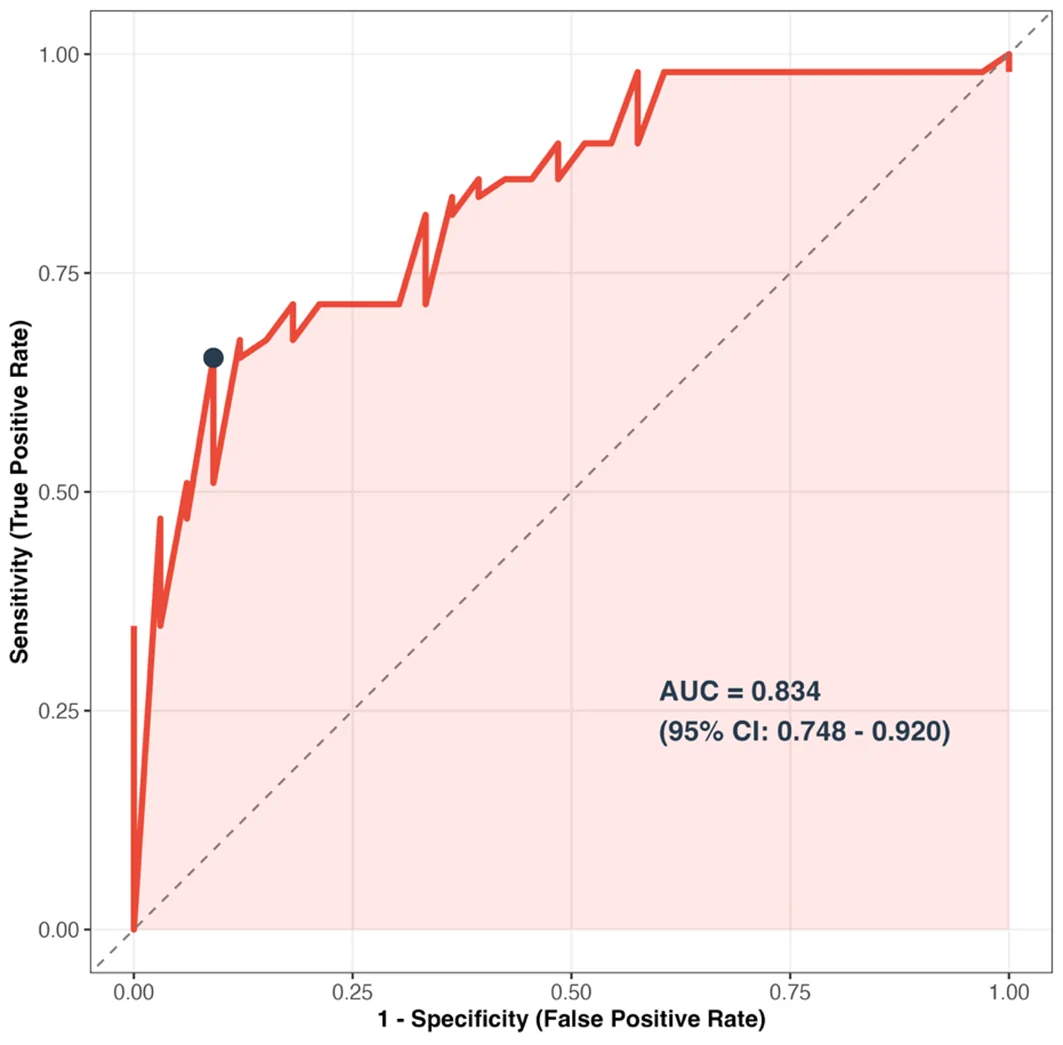
Receiver operating characteristic (ROC) curve illustrating the performance of the logistic regression model in predicting hypercholesterolaemia. The ROC curve depicts the internal discriminatory performance of a multivariable logistic regression model that incorporates thyroid function parameters, anti-TPO antibody levels, and CIMT for predicting high total cholesterol (TC >200 mg/dL). The red curve represents the model’s true-positive rate (sensitivity) as a function of the false-positive rate (1 - specificity) across all classification thresholds. The diagonal dashed line represents chance performance (AUC = 0.50). The model achieved an area under the curve (AUC) of 0.834 (95% confidence interval, 0.748-0.920), indicating acceptable internal discrimination. Because the model was not externally validated and was developed in a modest single-centre cohort, this result should be interpreted as exploratory model performance rather than proof of clinical utility.

To identify independent predictors of hypercholesterolaemia, we constructed a multivariable binary logistic regression model (**Tables 5**, **6**, **Figures 4**, **5**) incorporating age (per year), sex (male), CIMT (per cm), and log10-transformed TSH and Anti-TPO antibodies as candidate predictors. Log transformations were applied to TSH and Anti-TPO because of their non-normal distributions and anticipated nonlinear relationships with the cholesterol outcome.

**Table 5:** Multivariable logistic regression results.

Variable	OR	95% CI lower	95% CI upper	OR (95% CI)	P-value	Significance
Intercept	3.884	0.017	1304.269	3.884 (0.017 - 1304.269)	0.6307	
Age (per year)	0.911	0.845	0.973	0.911 (0.845 - 0.973)	0.0089	**
Male gender	1.413	0.453	4.637	1.413 (0.453 - 4.637)	0.5555	
CIMT (per cm)	0.000	0.000	118574.559	0.000 (0.000 - 118574.559)	0.1944	
Log_10_(TSH)	1.243	0.428	3.774	1.243 (0.428 - 3.774)	0.6904	
Log_10_(Anti-TPO)	13.629	1.174	190.489	13.629 (1.174 - 190.489)	0.0421	*

Outcome: High total cholesterol (TC > 200 mg/dL).

Significance codes: *** p<0.001, ** p<0.01, * p<0.05, † p<0.1.

**Table 6:** Model fit statistics.

Model	AIC	BIC	McFadden_R2	Nagelkerke_R2
Model 1a (Raw)	92.30	106.74	0.2735	0.4166
Model 1b (Log-transformed)	93.92	108.36	0.2589	0.3980
Model 1c (TPO Binary)	97.98	112.42	0.2221	0.3495

Model 1b AUC: 0.834 (95% CI: 0.748 - 0.920).

#### Model performance

The log-transformed Model 1b showed an internally assessed AUC of 0.834 (95% CI: 0.748-0.920) for distinguishing patients with and without hypercholesterolaemia, indicating acceptable internal discrimination. Model fit statistics were as follows: McFadden pseudo-R2 0.26, Nagelkerke R2 0.39, AIC 93.9 and BIC 108.4. Model 1a had a slightly lower AIC (92.3), whereas Model 1c had a higher AIC (97.9). **Figure 4** presents descriptive LOESS-smoothed plots of the probability of high total cholesterol across log10(TSH), log10(Anti-TPO) and CIMT. In the multivariable logistic regression model (**Table 5**), log10(TSH) was not independently associated with hypercholesterolaemia (p = 0.6904), whereas log10(Anti-TPO) was associated with hypercholesterolaemia (p = 0.0421). Because the Anti-TPO estimate had a wide 95% CI and the model was developed in a modest single-centre cohort without external validation, these findings should be interpreted as exploratory internal model results rather than as a ready-to-use clinical prediction tool.

## Discussion

### Integration of findings: mechanisms of thyroid dysfunction-associated atherosclerosis

The present study suggests that thyroid dysfunction, particularly overt hypothyroidism, is associated with early atherosclerotic markers as evidenced by elevated CIMT and lipid abnormalities. The positive overall correlation between TSH and CIMT is compatible with a thyroid-status-related gradient in vascular measures; however, because this was a cross-sectional analysis and within-group correlations were modest, the relationship should not be interpreted as proof of a nonlinear dose-response effect. These findings remain consistent with previous reports linking hypothyroid states with increased CIMT, while also emphasising the need for longitudinal studies to assess whether thyroid optimisation modifies vascular outcomes (13, 14).

The Anti-TPO-CIMT correlation supports the possibility that thyroid autoimmunity may contribute to vascular risk alongside hormone deficiency, but this interpretation should be approached with caution. Mechanistic studies suggest that thyroid autoimmunity can be accompanied by chronic immune activation and systemic inflammation. In this cohort, overt hypothyroid patients had both higher Anti-TPO levels and higher CIMT, whereas subclinical hypothyroid patients had less marked CIMT changes. This pattern is consistent with contributions from hormone deficiency, autoimmunity, and disease duration, but the present data cannot distinguish among causal pathways (8, 15).

### Lipid profiles as partial mediators of atherosclerotic risk

While traditional lipid parameters showed expected directional changes across thyroid states, the modest strength of individual lipid-CIMT correlations and the uniformity of LDL-C across groups suggest that dyslipidaemia alone may not fully account for CIMT differences in this cohort. Because current statin and lipid-lowering therapy users were excluded, the observed LDL-C pattern is unlikely to be explained by ongoing lipid-lowering treatment. Other factors, including age distribution, unmeasured lifestyle factors, duration of thyroid disease, endothelial dysfunction, oxidative stress and inflammation, may have contributed to vascular differences (9).

### Sex differences and age-related considerations

Epidemiological observations show that postmenopausal women experience accelerated atherosclerosis following oestrogen withdrawal, and that thyroid dysfunction may disproportionately affect cardiovascular risk in this population (16, 17). The mean cohort age of 47± 9 years indicates a middle-aged population in which both thyroid dysfunction and atherosclerosis are prevalent, potentially limiting generalisability to younger populations or older cohorts in which thyroid dysfunction-atherosclerosis associations may differ (18, 19).

### Anti-TPO as a potential marker of cardiometabolic risk

The multivariable logistic regression analysis suggests that Anti-TPO may be associated with hypercholesterolaemia after adjustment for age, sex, CIMT and log10(TSH). This observation is clinically interesting because it raises the possibility that thyroid autoimmunity may provide information beyond thyroid hormone status alone. However, the effect estimate was imprecise (95% CI: 1.174-190.489), and the result should therefore be interpreted as hypothesis-generating rather than definitive.

A cautious biological interpretation is that thyroid autoimmunity could contribute to lipid dysregulation through inflammatory pathways that are not fully captured by TSH alone. Thyroid hormone replacement normalises thyroid hormone status, but it may not directly suppress the underlying autoimmune process. Prior studies have linked autoimmune thyroid disease with immune activation and dyslipidaemia (20, 21), while residual inflammatory risk can persist despite contemporary lipid-lowering strategies (22). The present study does not prove these mechanisms, and inflammatory biomarkers were not measured.

### Clinical translation: stratified risk assessment and therapeutic implications

The observed Anti-TPO association provides a rationale for further evaluation of anti-TPO-stratified cardiovascular risk assessment in thyroid-disordered populations. Current cardiovascular risk tools, including Framingham-based approaches and ASCVD risk estimation, do not incorporate Anti-TPO status (23, 24). Whether Anti-TPO improves risk prediction beyond established clinical, biochemical and imaging markers requires prospective validation and formal assessment of calibration, discrimination and net clinical benefit.

The potential therapeutic implications should be regarded strictly as hypotheses. If future studies confirm that inflammatory pathways mediate part of the thyroid autoimmunity-lipid association, trials or mechanistic studies could evaluate whether targeted anti-inflammatory approaches modify vascular or lipid outcomes in carefully selected patients. The present cross-sectional study does not support the clinical use of anti-TNF-alpha, IL-6 pathway inhibition or other immunomodulatory therapy for cardiovascular prevention in Anti-TPO-positive thyroid disease (25–27).

### Clinical implications and management perspectives

The findings have several cautious clinical and research implications. First, a comprehensive thyroid assessment, including Anti-TPO measurement, may help characterise risk in research cohorts of patients with thyroid dysfunction. Second, optimisation of thyroid status remains clinically important in overt thyroid disease. Third, the Anti-TPO findings should not be used to change routine cardiovascular risk-assessment practice until prospective external validation confirms incremental predictive value.

Multifactorial interventions addressing lipid abnormalities (with statins or ezetimibe), blood pressure control, glycaemic optimisation, and weight management may be required to accompany thyroid hormone optimisation for comprehensive cardiovascular risk reduction.

### Study strengths

This study possesses several methodological strengths: (1) comprehensive phenotyping of thyroid dysfunction across the full spectrum of overt and subclinical disease, enabling assessment across disease severity gradients; (2) simultaneous measurement of thyroid hormones (TSH, Free T3, Free T4), thyroid autoimmunity (Anti-TPO), and multiple vascular and lipid markers; (3) a prespecified multivariable modelling approach with model fit assessment (AIC, BIC, pseudo-R2) and ROC analysis; (4) presentation of descriptive predicted probability curves; and (5) distinction between univariate associations and exploratory multivariable estimates.

### Study limitations

Important limitations should be acknowledged. First, the cross-sectional design precludes causal inference about whether Anti-TPO contributes to cholesterol elevation or serves as a marker of an underlying inflammatory/metabolic phenotype. Second, the small sample size resulted in wide confidence intervals for odds ratio estimates, particularly for Anti-TPO (95% CI: 1.174-190.489), and limited statistical power for subgroup analyses. External validation in larger, independent cohorts is essential before clinical implementation of Anti-TPO-stratified risk algorithms. Third, the cohort was derived from a single Indian tertiary care centre, potentially limiting generalisability to other ethnic groups, geographic regions, and healthcare settings. Fourth, patients receiving thyroid supplementation, antithyroid medications, statins or other lipid-lowering therapy were excluded, which reduced treatment-related confounding but limits generalisability to treated thyroid-disease populations commonly seen in routine practice. Fifth, although thyroid and lipid parameters were measured using routine automated laboratory methods on the cobas^®^ 6000 analyser series platform, individual reagent lot numbers, assay batch identifiers, and detailed kit batch information were not retained. Sixth, inflammatory biomarkers (TNF-alpha, IL-6, high-sensitivity CRP) were not measured, precluding direct mechanistic validation of inflammation-mediated pathways. Seventh, additional atherosclerosis markers, including coronary artery calcium scoring, carotid plaque characterisation, and advanced vascular imaging, were not employed, thereby limiting comprehensive assessment of atherosclerotic disease burden. Eighth, the inverse age association in the hypercholesterolaemia model should be interpreted cautiously, as it likely reflects cohort composition and the older age of the subclinical hypothyroidism group rather than a true protective effect of ageing. In addition, because a formal *a priori* sample-size calculation was not performed, non-significant subgroup comparisons should not be interpreted as evidence of no association. The *post hoc* power assessment supports the main overall correlation analyses but does not mitigate the limited precision of subgroup and multivariable estimates.

## Conclusion

In summary, this exploratory single-centre study found that overt hypothyroidism was associated with early atherosclerotic changes and dyslipidaemia in an Indian adult cohort with thyroid dysfunction. Anti-TPO antibodies correlated with CIMT and were associated with elevated total cholesterol in an adjusted model; however, the wide confidence interval, modest sample size, and cross-sectional design mean that this finding should be interpreted with caution. Larger prospective studies with external validation, including treated and untreated patient populations and measurement of inflammatory biomarkers, are required to clarify whether Anti-TPO adds clinically meaningful information to cardiovascular risk assessment in thyroid disease.

## Data Availability

The raw data supporting the conclusions of this article will be made available by the authors, without undue reservation.
